# The potential roles of NAD(P)H:quinone oxidoreductase 1 in the development of diabetic nephropathy and actin polymerization

**DOI:** 10.1038/s41598-020-74493-z

**Published:** 2020-10-20

**Authors:** Sung-Je Moon, Jin Young Jeong, Jae-Hoon Kim, Dong-Hee Choi, Hyunsu Choi, Yoon-Kyung Chang, Ki Ryang Na, Kang Wook Lee, Chul-Ho Lee, Dae Eun Choi, Jung Hwan Hwang

**Affiliations:** 1 Laboratory Animal Resource Center, Korea Research Institute of Bioscience and Biotechnology (KRIBB), 125 Gwahak-ro, Yuseong-gu, Daejeon, 34141 Korea; 2 KRIBB School of Bioscience, Korea University of Science and Technology (UST), 217 Gajeong-ro, Yuseong-gu, Daejeon, 34113 South Korea; 3 Department of Nephrology, School of Medicine, Chungnam National University, 266 Munwha-ro, Jung-gu, Daejeon, Korea; 4 Clinical Research Institute, Daejeon St. Mary Hospital, Daejeon, South Korea; 5 Department of Nephrology, Catholic University of Korea, Seoul, South Korea

**Keywords:** Cell biology, Molecular biology, Diseases, Nephrology, Pathogenesis

## Abstract

Diabetic nephropathy (DN) is a major complication of diabetes mellitus. NAD(P)H:quinone oxidoreductase 1 (NQO1) is an antioxidant enzyme that has been involved in the progression of several kidney injuries. However, the roles of NQO1 in DN are still unclear. We investigated the effects of NQO1 deficiency in streptozotocin (STZ)-induced DN mice. NQO1 was upregulated in the glomerulus and podocytes under hyperglycemic conditions. NQO1 knockout (NKO) mice showed more severe changes in blood glucose and body weight than WT mice after STZ treatment. Furthermore, STZ-mediated pathological parameters including glomerular injury, blood urea nitrogen levels, and foot process width were more severe in NKO mice than WT mice. Importantly, urine albumin-to-creatinine ratio (ACR) was higher in healthy, non-treated NKO mice than WT mice. ACR response to STZ or LPS was dramatically increased in the urine of NKO mice compared to vehicle controls, while it maintained a normal range following treatment of WT mice. More importantly, we found that NQO1 can stimulate actin polymerization in an in vitro biochemical assay without directly the accumulation on F-actin. In summary, NQO1 has an important role against the development of DN pathogenesis and is a novel contributor in actin reorganization via stimulating actin polymerization.

## Introduction

One of the main complications of diabetes is diabetic nephropathy (DN), which most commonly leads to end-stage renal disease and develops in approximately 30% of patients with diabetes mellitus^1^. The common pathological features of DN include glomerular hypertrophy, deposition of extracellular matrix (ECM) proteins, expansion of mesangial matrix and glomerular basement membrane, and increased proteinuria early in disease development^2,3^. Although various components of the glomerulus including endothelial cells, mesangial cells, and podocytes have been involved in the progression of DN, podocyte loss is a hallmark of DN^4,5^. Podocyte injury is the mechanistic basis of proteinuria in DN and a critical indicator of its severity^6^.

Podocytes are highly differentiated kidney glomerulus cells that form the filtration system together with the endothelial cells in the capillaries and the glomerular basement membrane (GBM)^7,8^. These cells are composed of a cell body and primary, secondary, and tertiary foot processes (FP)^9^. The structure of FPs is maintained by a complex cytoskeleton network consisting of microtubules, intermediate filaments, and actin^10^. FPs form an interdigitating architecture linked with the external wall of the capillaries through a specialized cell junction known as the slit diaphragm (SD)^11,12^. In the early stages of DN, loss of FPs, defined as foot process effacement (FPE), is found in injured-podocytes^13^. FPE is the consequence of dysregulation of the actin cytoskeleton, suggesting a direct link between proteinuria and actin cytoskeleton dynamics^14^. Furthermore, dysregulated podocyte cytoskeleton or loss of integrity of SD is a common pathological finding in DN^14^.

Following podocyte damage, the FPs of podocytes are recovered through a distinct re-organization of the actin cytoskeleton^15^. It can be achieved only by induction of actin polymerization in the vicinity of the membrane. Both FPE and reformation require distinct signals into common hubs (a SD and focal adhesions) that control assembly and dis-assembly of actin^16^. Small GTPases such as RhoA, Cdc42, and Rac1 are the main contributors of actin cytoskeleton remodeling by acting as molecular switches^17^. Accumulating evidence has suggested that they are also involved in the pathogenesis of podocyte injuries. Modulation of RhoA signaling was able to alleviate podocyte injury murine chronic kidney disease model^18,19^. Expression of dominant-negative RhoA in mice led to loss of stress fiber formation in podocytes, resulting in proteinuria^20^. Thus, the balance of RhoA signaling is imperative for normal podocyte physiology. A genetic animal model for Rac1 displayed resistance and sensitivity to puromycin induced-podocyte injury and chronic hypertensive glomerular sclerosis injury, respectively^21^. Podocyte-specific Cdc42 knockout mice exhibited severe proteinuria, FPE, glomerulosclerosis, and congenital nephropathy^21,22^. Furthermore, mutations in human genes that interfere with Rho GTPase signaling have been identified in nephrotic diseases including focal segmental glomerulosclerosis (FSGS) and DN^23^. However, the mechanisms regulating podocyte cytoskeleton during FPE are still unknown.

NAD(P)H:quinone oxidoreductase 1 (NQO1 1; DT-diaphorase) is a cytosolic two-electron reductase activated by various quinone compounds^24^. It has been well known as an antioxidant enzyme transcribed by nuclear factor E2-related factor 2 (Nrf2), a key regulator of the antioxidant response^25,26^. Our recent studies have suggested that NQO1 has a protective role against renal ischemia/reperfusion injury and cisplatin-induced nephrotoxicity in mice models^27,28^. In both these kidney-injured models, NQO1 knockout (NKO) mice showed increased tubular damage, apoptosis, and oxidative stress via the regulation of cellular NADPH/NADP redox system. In contrast, activation of NQO1 by beta-lapachone (βL) improved renal dysfunction and reduced tubular cell damage, oxidative stress, and apoptosis^27^. Previously, Zappa and colleagues have found specific high expression of NQO1 in podocytes^29^. However, the roles of NQO1 in podocytes during development of DN is completely unclear. In the current study, we investigated its roles in the podocytes of STZ-induced DN in a mouse model.

## Results

### Upregulation of NQO1 in the kidney glomerulus of mice with diabetic nephropathy and in podocytes under high glucose condition

**Figure 1:**
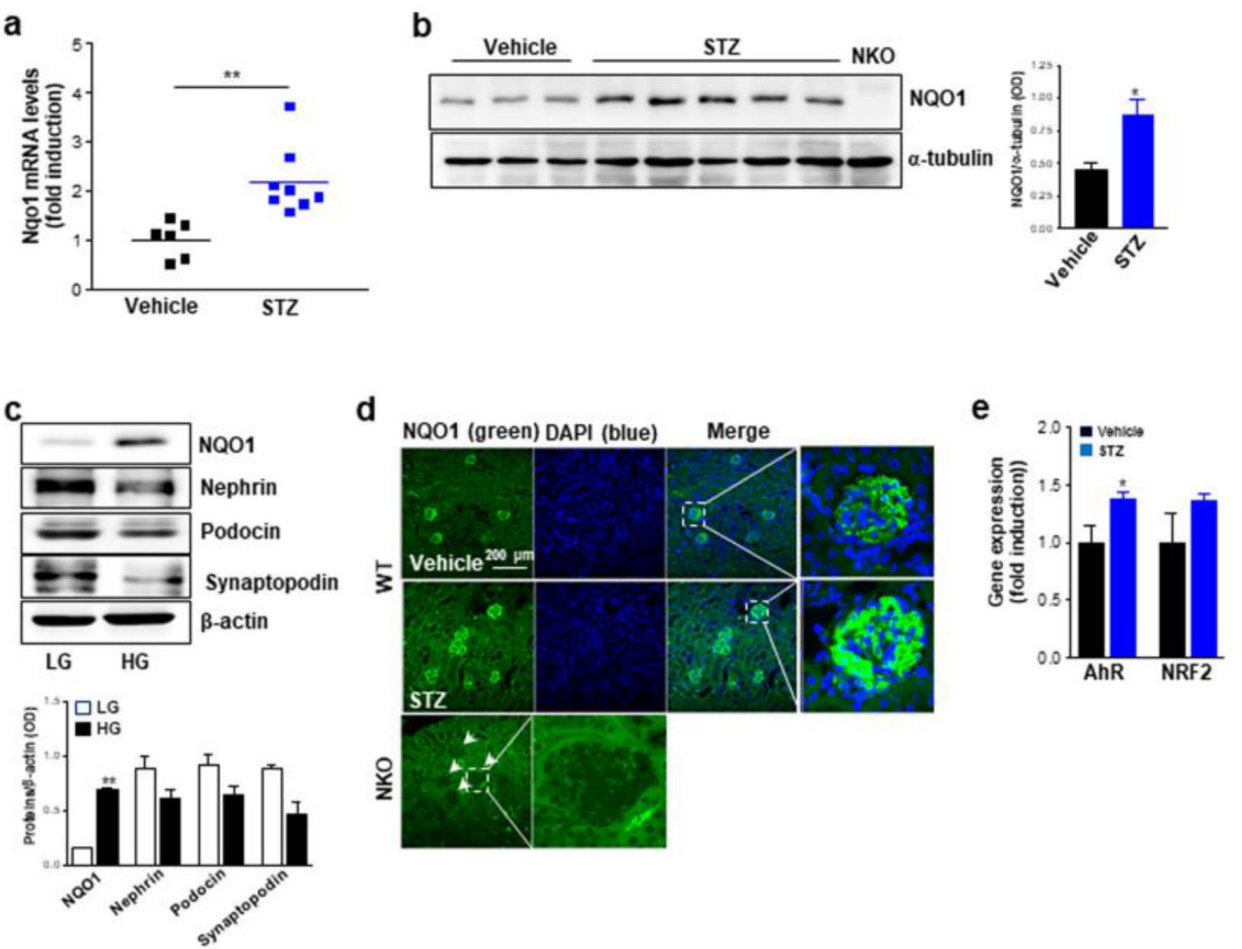
mRNA and protein expression of NQO1 are upregulated in hyperglycemic conditions. (**a**, **b**) mRNA and protein expression levels of NQO1 in the kidney tissues from mice treated with vehicle (n = 6) or STZ (n = 8) for 8 weeks were measured by qRT-PCR and western blot analyses, respectively. Original blot image is presented in Fig. S1. The optical density of NQO1 protein was normalized by α-tubulin. All values are means ± SEM. *P < 0.05 or **P < 0.01. (**c**) Alterations of NQO1 and podocyte-specific proteins were estimated by western blot analysis of lysates from cells maintained in low-glucose or high-glucose conditions. Original blot image is presented in Fig. S2. The optical density of proteins is normalized by β-actin and representative of two independent experiments. All values are means ± SEM. **P < 0.01. (**d**) Fluorescence image (× 20 magnification) of NQO1 in representative kidney sections from WT mice and NKO mice using NQO1-specific antibody. Green is NQO1 and blue indicates DAPI. Scale bar is 200 μm. (E) AhR and NRF2 mRNA levels in the kidney tissues from mice treated with vehice (n = 3) or STZ (n = 5) for 8 weeks were estimated by qRT-PCR. *P < 0.05. Protein levels were analyzed by Image J software program (1.43u, https://imagej.nih.gov).

We first estimated the expression levels of NQO1 in vivo and in vitro under high glucose condition. NKO and WT mice in the C57BL/6N background were used to induce DN by administration of STZ. Protein and mRNA levels of NQO1 were significantly induced in the kidney tissues at 8 weeks after STZ treatment compared to those of vehicle treated mice (Fig. 1A,B). To determine the expression of NQO1 in a hyperglycemic situation, podocytes were cultured in medium containing a low glucose or high glucose and compared the levels of several proteins that has been shown to be altered in DN. The protein levels of nephrin, podocin, and synaptopodin were decreased in cells cultured in high glucose compared to those cultured in low glucose condition, while NQO1 protein was significantly increased in the high glucose condition (Fig. 1C). To determine NQO1 distribution in the kidney tissue, we stained the kidney tissues with an antibody specific for NQO1. Consistent with a previous human study^29^, NQO1 protein was mainly detected in the kidney glomerulus and fluorescence intensity was higher in the kidney tissue from mice treated with STZ compared to those of mice treated with vehicle (Fig. 1D). These data suggest that NQO1 expression is altered by diabetic conditions in vitro and in vivo*.* Nuclear factor erythroid 2 (NFE2)-related factor 2 (NRF2) and arylhydrocarbon receptor (AhR) have been known as two major transcription factors for NQO1^30^. Interestingly, AhR mRNA expression was significantly up-regulated in the kidney tissues from STZ-treated mice compared to vehicle-treated mice, while Nrf2 mRNA expression was statistically not significant (Fig. 1E). This result provides that AhR might be an important transcriptional regulator of NQO1 in STZ-induced DN.

### Aggravated renal injury in NKO mice

**Figure 2:**
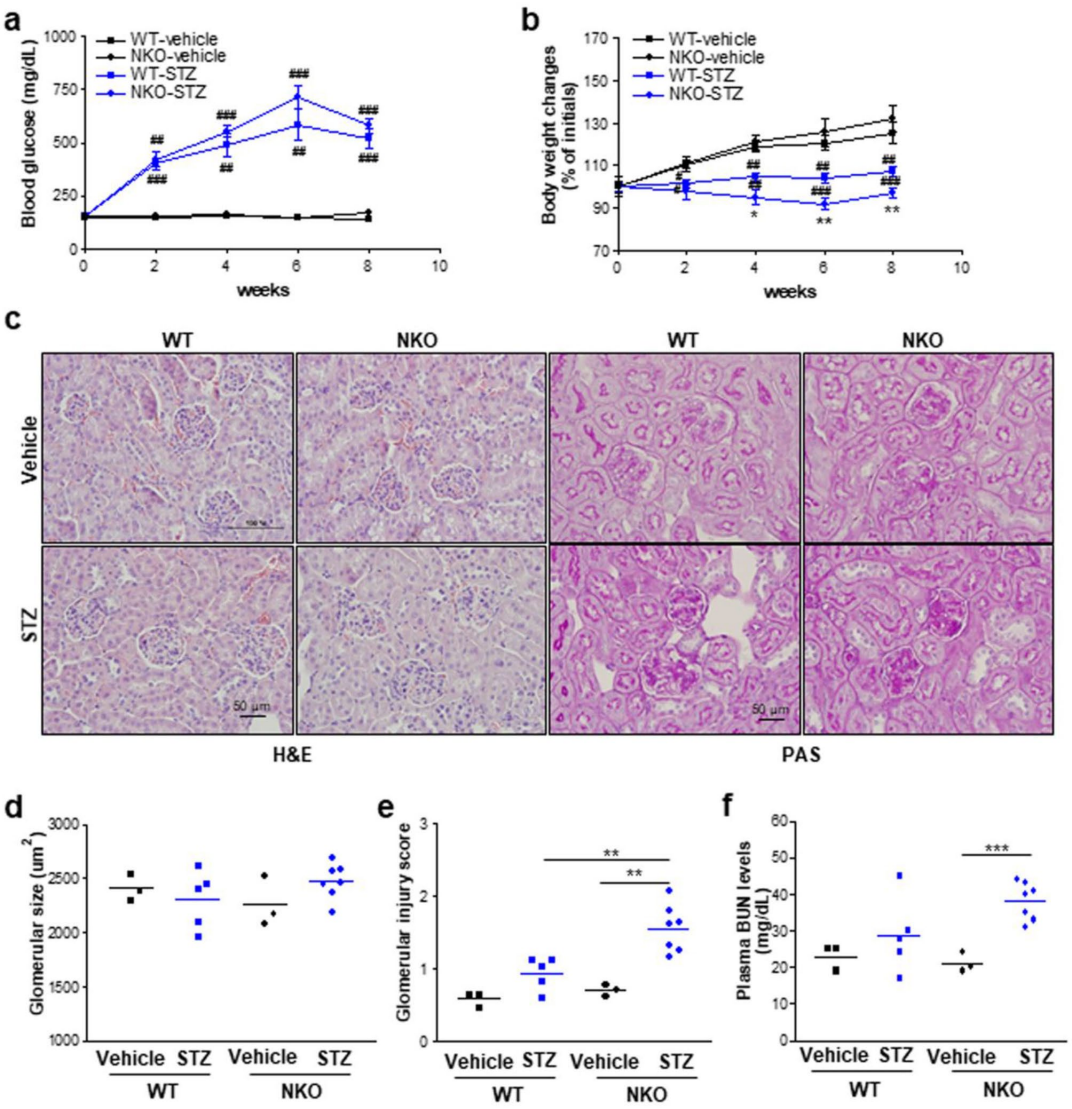
NQO1 deficiency lead to aggravated kidney injury in STZ-induced DN mice. (**a**, **b**) Blood glucose and body weight changes of WT mice (vehicle, n = 3; STZ, n = 5) and NKO mice (vehicle, n = 3; STZ, n = 7) were monitored during the indicated periods. All values are means ± SEM. *P < 0.05 or **P < 0.01 vs WT; ^#^*P* < 0.05, ^##^*P* < 0.01, or ^##^*P* < 0.001 vs vehicle. (**c**) Representative kidney sections were stained with H&E (× 20 magnification) or Periodic acid-Schiff (PAS, × 20 magnification). Scale bar is 50 μm. (**d**) Glomerular size was determined in the H&E stained kidney sections from WT mice and NKO mice. (**e**) Glomerular injury score was estimated in the PAS stained kidney sections. (**f**) Plasma BUN levels. Data are representative of at least two independent experiments. *P < 0.05, **P < 0.01 or ***P < 0.001.

We have previously shown^31^ that NKO mice displayed increased pancreatic β-cell apoptosis and subsequently hyperglycemia, impaired glucose clearance rate, and lower plasma insulin levels following low dose STZ injections. In the current study, we observed that STZ induced a sustained increase in serum glucose levels in both mice from 1 to 8 weeks, with the maximum concentration of serum glucose at 6 weeks (Fig. 2A). However, STZ-induced blood glucose upregulation was higher in NKO mice than WT mice but the weight reduction in NKO mice was comparable to that in WT mice under the diabetic conditions (Fig. 2A,B). To investigate whether NQO1 is associated with renal injury in DN, we estimated the renal damage by hematoxylin & eosin (H&E) and Periodic acid-Schiff (PAS) staining of kidney tissues (Fig. 2C). No significant changes in glomerular size were observed in the kidney tissues from all groups (Fig. 2D). In WT mice, STZ induced minor glomerular damages, but not significant compared with the vehicle-treated group (Fig. 2E). Interestingly, the STZ-induced glomerular injury in NKO mice was significantly higher than that in WT mice (Fig. 2E). Furthermore, STZ-induced glomerular damages such as glomerular hypertrophy and mesangial expansion, were higher in the kidney from NKO than in WT mice (Fig. 2E). In addition, STZ-induced serum BUN increases were only observed in the NKO mice (Fig. 2F). The data suggest that knockout of NQO1 leads to increased susceptibility to STZ-induced glomerular injury.

### Exacerbated podocyte injury in NKO mice after administration of STZ

**Figure 3:**
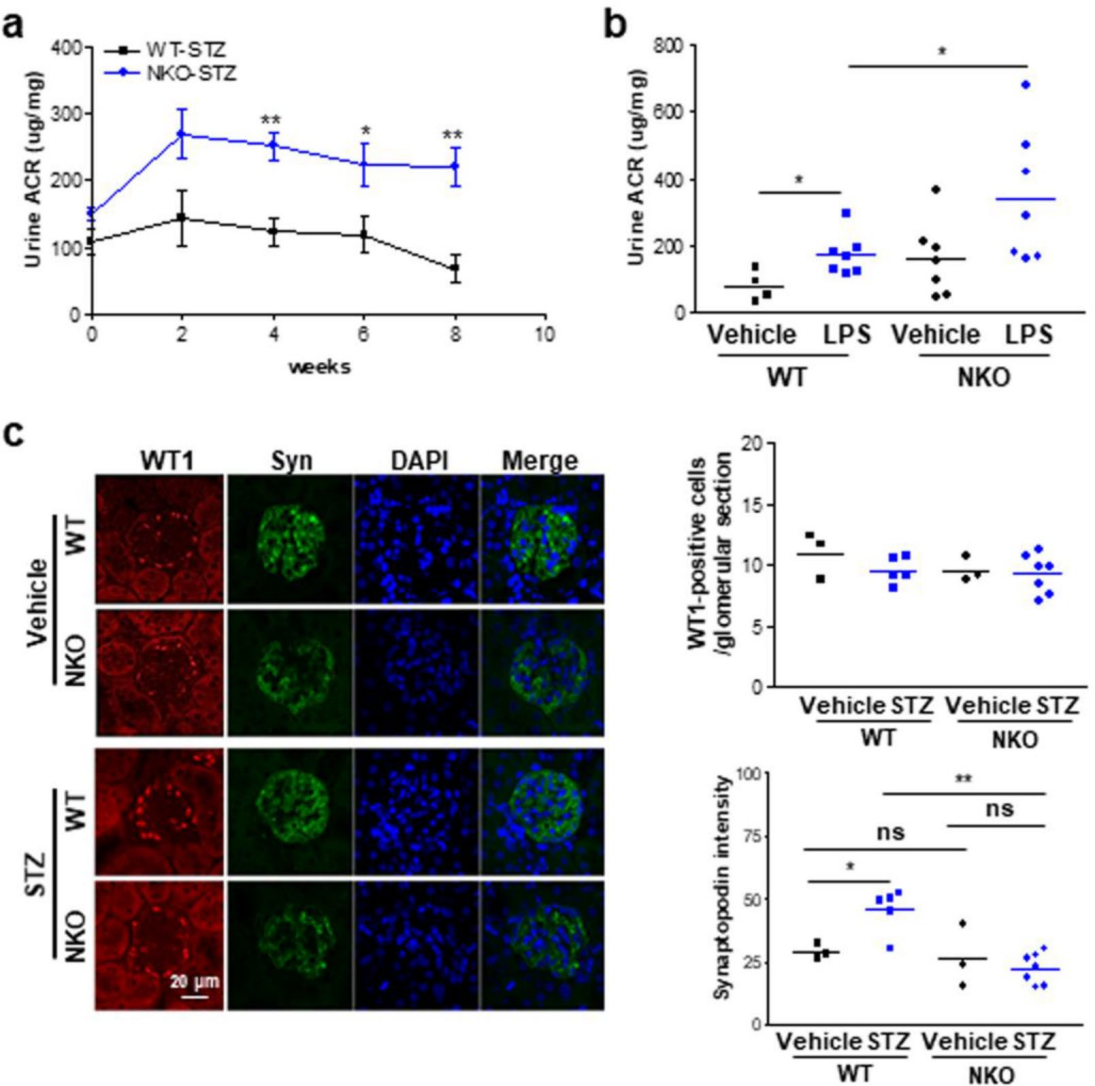
NQO1 deficiency results in increased ACR and abnormal synaptopodin localization in response to STZ or LPS. (**a**) Urine albumin-to-creatinine ration (ACR) of WT mice and NKO mice at 0 (WT, n = 6; NKO, n = 6), 2 (WT, n = 3; NKO, n = 4), 4 (WT, n = 7; NKO; n = 7), 6 (WT, n = 5; NKO, n = 7), 8 weeks (WT, n = 5; NKO, n = 7) after STZ treatment was measured. *P < 0.05, **P < 0.01. (**b**) Urine ACR of WT mice (vehicle, n = 4; LPS, n = 7) and NKO mice (vehicle, n = 6; LPS, n = 7) is shown at 1 day post-LPS administration (15 mg/kg). *P < 0.05. (**c**) The numbers of WT-1 positive cell and the distribution of synaptopodin were estimated by immunostaining (× 60 magnification) using specific antibodies against WT1 (red) and synaptopodin (green). Scale bar is 20 μm. Fluorescence intensity of synaptopodin was analyzed using ImageJ software program (1.43u, https://imagej.nih.gov). *P < 0.05, **P < 0.01.

Albuminuria and podocyte loss are the best indicators of chronic kidney disease in patients with diabetes. Therefore, we monitored the albumin/creatinine ratio (ACR) in urine after STZ treatment. STZ treatment resulted in upregulation of urine ACR levels in NKO mice at 0, 2, 4, 6, and 8 weeks, while WT mice treated with STZ maintained normal ACR levels during the experiment (Fig. 3A). Next, we further measured the urine ACR levels in both mice treated with lipopolysaccharide (LPS) for 1 day to investigate glucose dependency (Fig. 3B). LPS treatment led to significantly increased urine ACR levels in WT mice compared with WT-vehicle (Fig. 3B). Interestingly, NKO mice exhibited significant higher ACR levels in the urine than WT mice after LPS treatment, suggesting that glomerular injury in NKO mice can occur in a glucose-independent manner (Fig. 3B). To assess the effect of NQO1 ablation on podocyte integrity, we stained kidney tissues using an antibody against Wilm’s tumor 1 (WT1), a master transcription factor specifically expressed in podocytes. The number of podocytes was not different in between the four groups (Fig. 3C). However, fluorescence intensity of synaptopodin, a podocyte-specific marker that binds to actin filaments, was significantly upregulated in podocytes of STZ-treated WT mice compared to vehicle-treated WT mice, while that in NKO mouse podocytes was not altered (Fig. 3C). These results indicate that NKO mice have functionally abnormal podocytes.

### Ultramicroscopic changes in the kidney from WT mice and NKO mice

**Figure 4:**
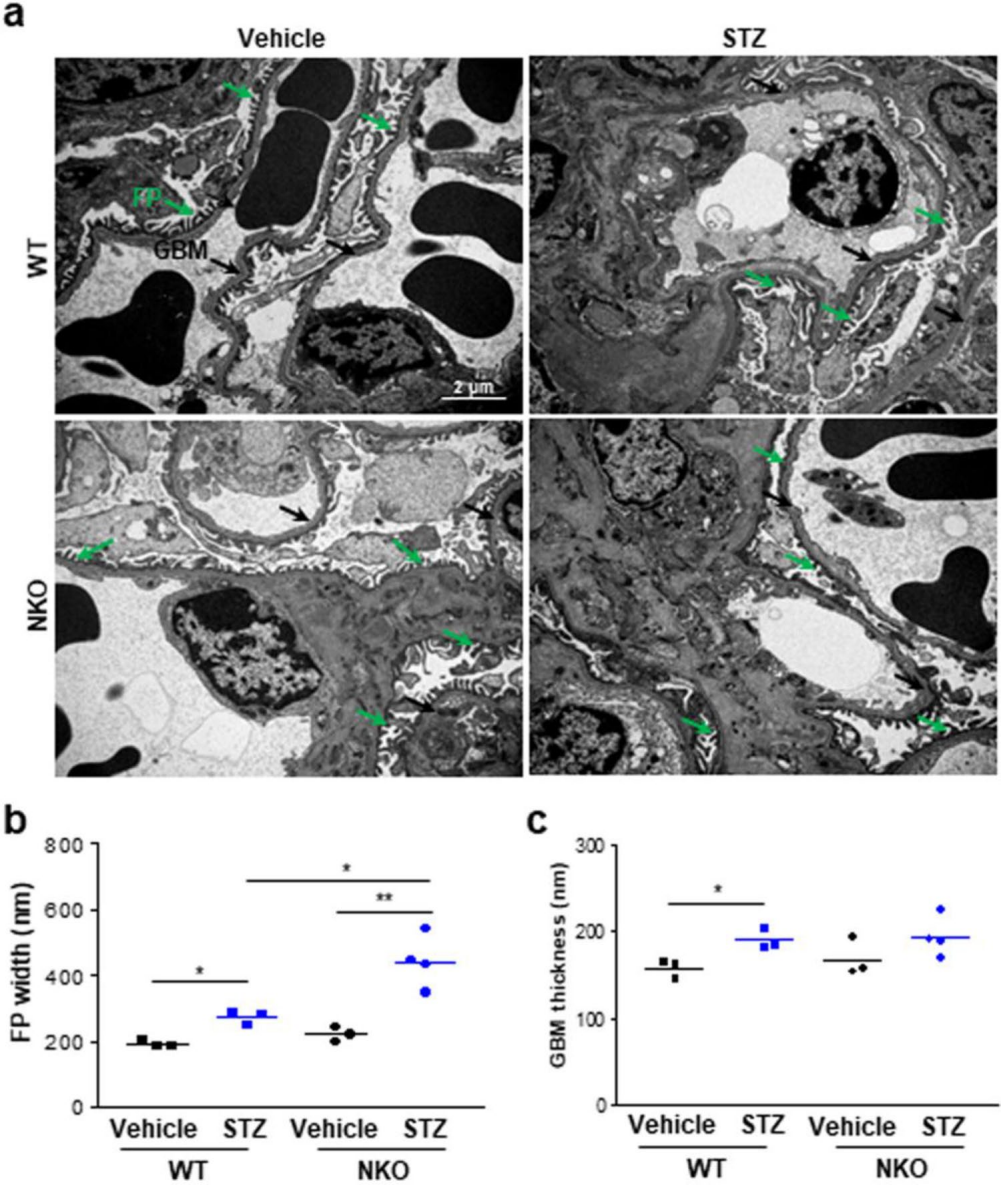
NKO mice exhibit increased foot process width in the healthy and disease conditions compared with WT mice. (**a**) Representative ultrastructural images of WT and KO mice treated with vehicle or STZ were obtained by transmission EM to show changes in podocyte structure. FP (green arrow) indicates a foot process and GBM (black arrow) is a glomerular basement membrane. Scale bar is 2 μm. (**b**) Quantitation of FP width and (**c**) GBM thickness of WT mice (vehicle, n = 3; STZ, n = 3) and NKO mice (vehicle, n = 3; STZ, n = 4) were determined in the TEM images. All values are means ± SEM. *P < 0.05 or **P < 0.01.

FPE is commonly found in the glomerulus of mice with DN. To estimate the effect of NQO1 knockout on FPE, we analyzed the ultramicroscopic alterations of the DN by transmission electron microscope (TEM). FP width was moderately, but significantly, increased in WT mice at 8 weeks after STZ treatment (Fig. 4A,B). Importantly, STZ-induced FP failure was stronger in NKO mice than WT mice, as evidenced by the analysis of FP width (Fig. 4A,B). However, although STZ treatment led to upregulation of glomerular basement membrane (GBM) thickness compared to vehicle-groups of both mice, there were no significant differences between NKO and WT mice (Fig. 4A,C) after STZ treatment. These data suggest that NQO1 plays a critical role in maintaining homeostasis of FPs in podocytes of mice with DN.

### ROS are not major mediators of STZ-induced DN pathogenesis in NKO mice

**Figure 5:**
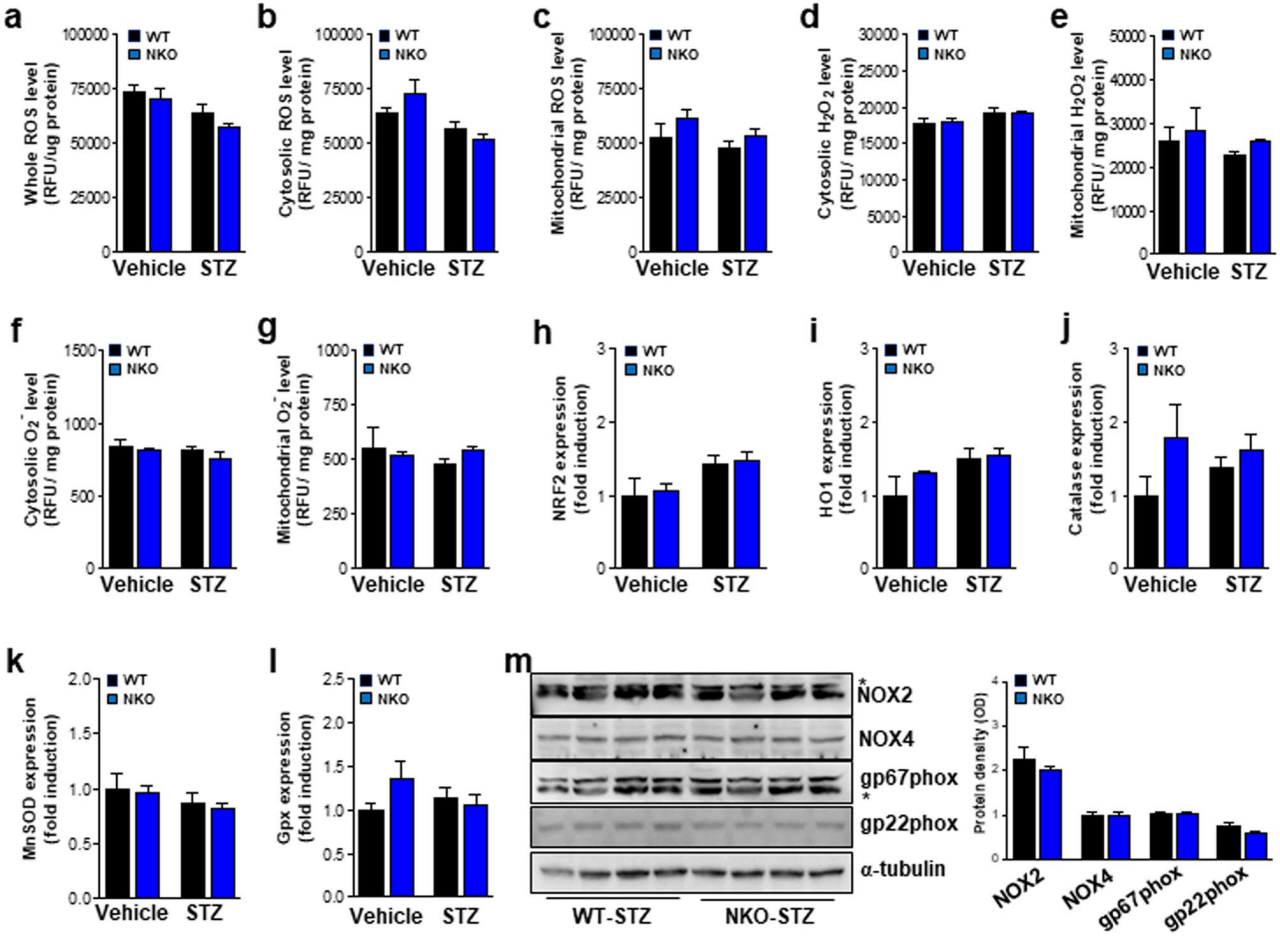
ROS production did not differ between WT mice and KO mice. Total ROS levels of whole (**a**), cytosolic fraction (**b**), or mitochondrial fraction (**c**) from kidney tissues of WT mice (vehicle, n = 3; STZ, n = 5) and NKO mice (vehicle, n = 3; STZ, n = 7) were measured by using DCF-DA. H_2_O_2_ levels of in the cytosolic fraction (**d**) or mitochondrial fraction (**e**) from kidney tissues of both groups. O_2_^-^ levels in the cytosolic fraction (**f**) or mitochondrial fraction (**g**). Renal mRNA levels of gene related with ROS such as NRF2 (**h**), HO1 (**i**), catalase (**j**), MnSOD (**k**), and Gpx (**l**) in WT mice (vehicle, n = 3; STZ, n = 5) and NKO mice (vehicle, n = 3; STZ, n = 7). (M) Protein levels of NOX family were estimated by western blot analysis and quantified using ImageJ software program (1.43u, https://imagej.nih.gov). *Indicated non-specific band. Original blot image is presented in Fig. S3.

We have previously^27^ observed that NKO mice displayed increased ROS generation and accelerated renal failure in a renal ischemia/reperfusion model and increased ROS production has been shown to be a critical contributor to the pathology of patients with DN. Therefore, ROS production was assessed in kidney tissues from both mice treated with vehicle or STZ. No upregulation of total ROS production was observed in whole tissues, cytosolic or mitochondrial fractions, in NKO and WT mice after STZ treatment (Fig. 5A–C). To investigate more closely the effect of NQO1 deficiency on STZ-induced ROS production, we measured H_2_O_2_ and O_2_^-^ in the cytosolic or mitochondrial fractions of kidney from NKO mice and WT mice. In line with the effect on the total ROS production, no significant differences were observed between NKO and WT mice treated with either vehicle or STZ (Fig. 5D–G). We also estimated the expression levels of NRF2, heme oxygenase 1 (HO1), and antioxidant enzymes such as catalase, superoxide dismutase (SOD), and glutathione peroxidase (Gpx). However, we did not observed any difference at expression levels of these genes between WT and NKO (Fig. 5H–L). In previous our study^27^, we showed that NQO1 inhibits NADPH oxidase (NOX)-derived ROS production in the renal ischemia/reperfusion injury animal model. Our western blot data showed no significant NOX protein levels in both groups after STZ treatment (Fig. 5M). These data suggest that ROS is not an important mediator or the underlying mechanism for the development of STZ-mediated renal pathogenesis in NKO mice.

### NKO mice showed dysregulated actin cytoskeleton integrity in the glomerulus.

**Figure 6:**
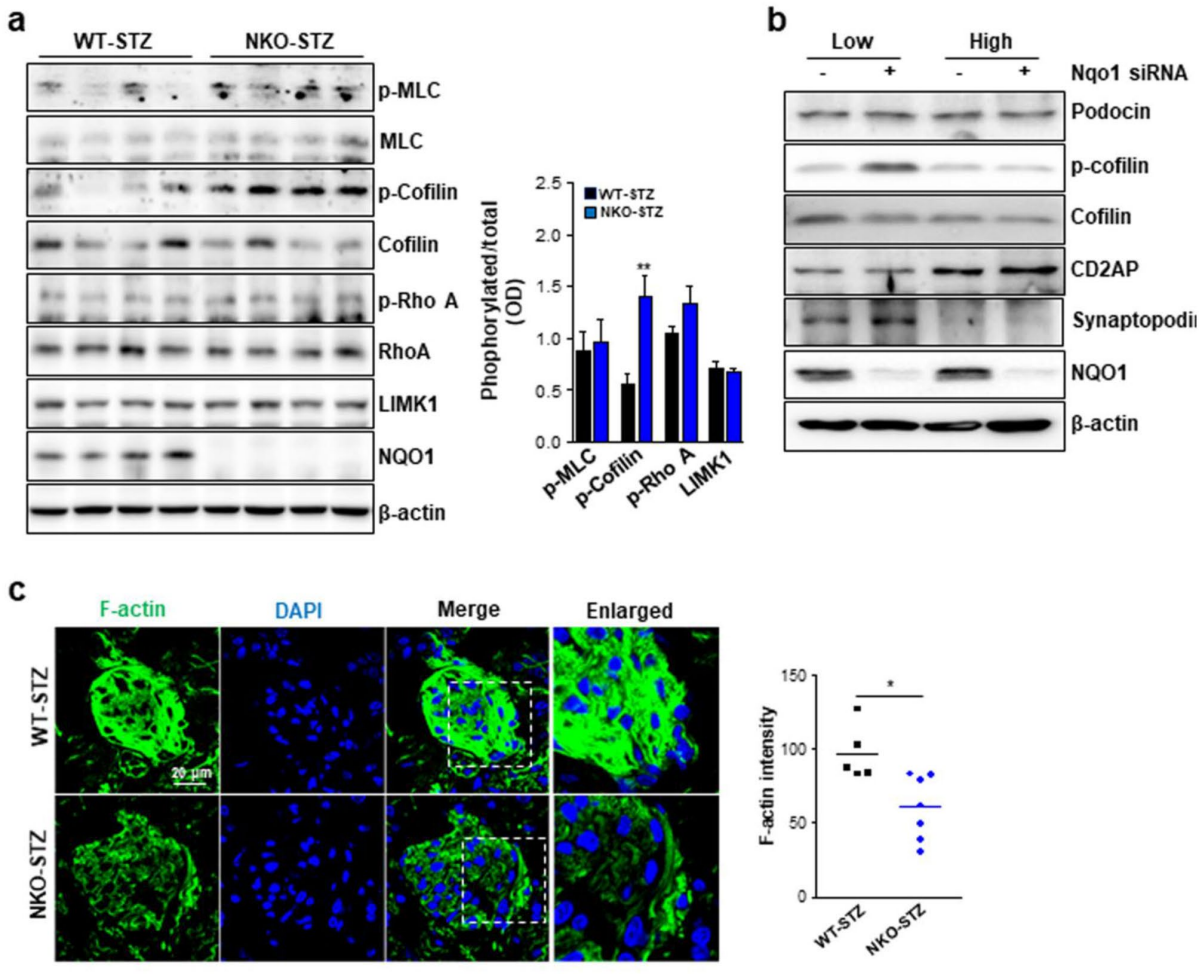
NKO mice show altered levels of proteins related with actin regulation and abnormal F-actin architecture. (**a**) Phosphorylation levels of proteins involved in reorganization of actin filament were assayed by western blot using specific antibodies against the indicated proteins. Original blot image is presented in Fig. S4.Quantitation of each band intensity was performed by ImageJ program (1.43u, https://imagej.nih.gov). **P < 0.01. (**b**) Podocytes were treated with scrambled siRNA or NQO1 siRNA and then cultured in low-glucose and high-glucose conditions. Original blot image is presented in Fig. S5. Phosphorylation levels of the indicated proteins were analyzed by western blot assay. (**c**) Representative fluorescent immunohistochemistry images (× 60 magnification) for F-actin (green). Scale bar is 20 μm. F-actin intensity was measured by ImageJ program (1.43u, https://imagej.nih.gov). *P < 0.05.

Based on above immunostaining for synaptopodin that is an F-actin binding protein, we concluded that NKO mice have a dysregulated actin cytoskeleton in podocytes. Therefore, to investigate our hypothesis, we compared phosphorylation of several proteins related in actin regulation such as MLC, cofilin, and RhoA. Interestingly, phosphorylation of cofilin, an actin-binding protein, which is involved in actin filament disassembly, was significantly upregulated in the kidney of STZ-treated NKO mice compared to that of STZ-treated WT mice, while no differences in the phosphorylation of MLC and RhoA were observed in both NKO and WT mice (Fig. 6A). LIM kinases has been well known as a major upstream kinase for cofilin. However, LIMK1 level in the kidney from NKO mice was similar to that of WT mice (Fig. 6A). To validate this data, we silenced the NQO1 in podocytes and performed western blot analysis of protein lysates from cells cultured under low-glucose or high-glucose conditions. NQO1 protein was efficiently downregulated by treatment with the siRNA for NQO1 (Fig. 6B). Consistent with the in vivo data, phosphorylation of cofilin was increased in NQO1 silenced podocytes under low-glucose condition, while was not affected by the high-glucose condition (Fig. 6B). In this experiment, we observed that synaptopodin was increased in NQO1 silenced podocytes cultured under low-glucose conditions (Fig. 6B). To investigate whether NQO1 is involved in the regulation of actin cytoskeleton integrity, we stained kidney tissues with ActinGreen 488 to detect the F-actin. The fluorescence intensity of F-actin was significantly lower in the kidneys from NKO mice than from WT mice (Fig. 6C). These results suggest that NQO1 can regulate actin cytoskeleton integrity in podocytes of mice with DN.

### Direct regulation of actin polymerization by NQO1 protein in vitro

**Figure 7:**
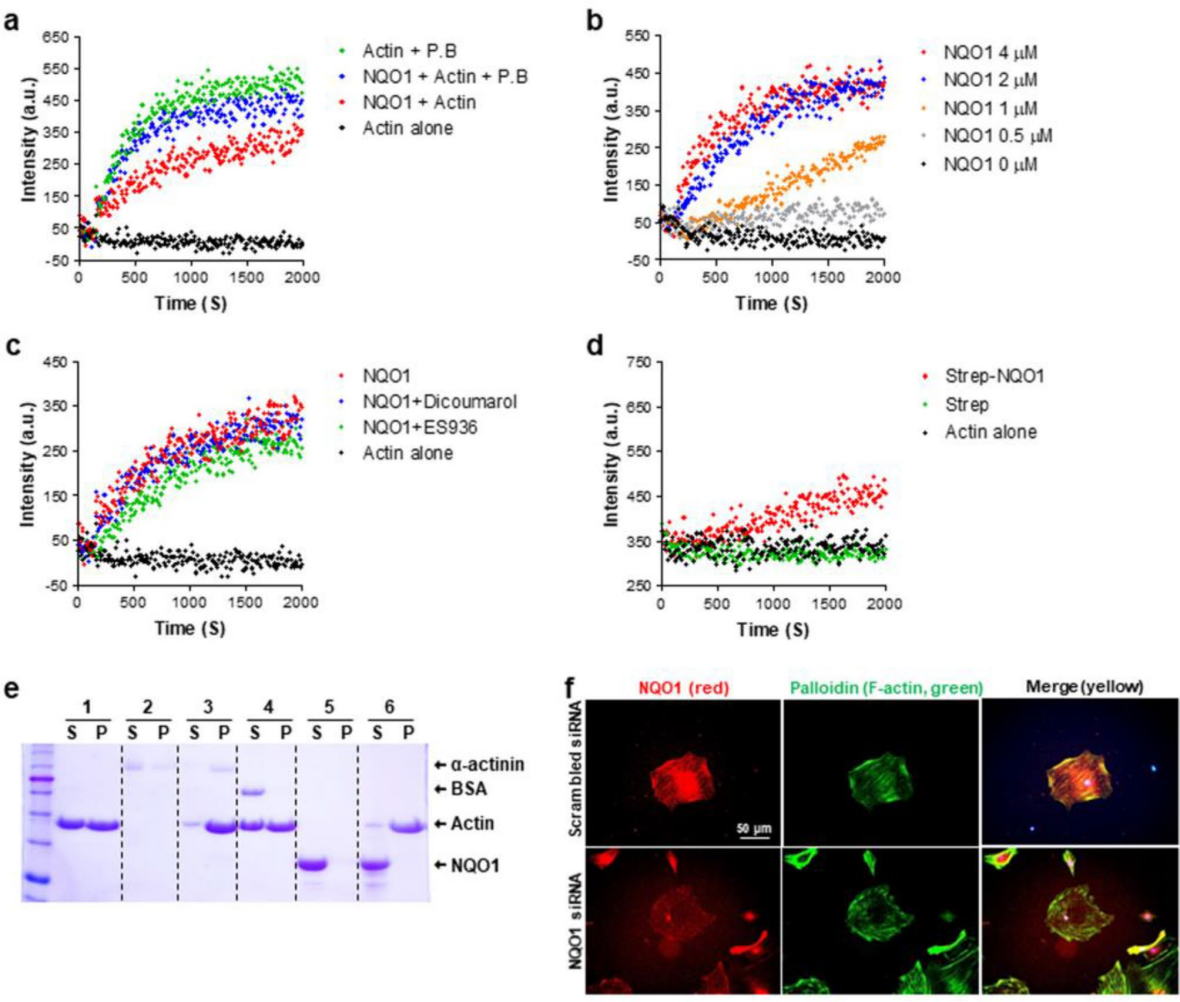
NQO1 protein stimulates action polymerization in vitro and accumulates with F-actin. (**a**) Pyrene actin assay to examine the involvement of recombinant human NQO1 in actin polymerization was performed in various combinations, actin alone (black), actin + polymerization buffer (PB, green), actin + NQO1 (2 μM, red), or actin + NQO1 + PB. PB used as a positive control for actin polymerization. (**b**) NQO1 effects dose-dependently actin polymerization. (**c**) The effects of two NQO1 specific inhibitor (dicoumarol, blue and ES936, green) on NQO1-mediated actin polymerization. (**d**) NQO1 protein (40 μM) purified in HEK293T cells was used in the actin polymerization reaction. (**e**) Association of NQO1 with F-actin was determined by an actin co-sedimentation assay. The supernatant (S) and pellet (P) fractions of each condition (Lane 1, F-actin alone; Lane 2, α-actinin alone; Lane 3, α-actinin and F-actin for positive control; Lane 4, BSA and F-actin for negative control; Lane 5, 20 μM NQO1 alone; Line 6, 20 μM NQO1 and F-actin) were separated on a 12% SDS polyacrylamide gel electrophoresis and stained with Coomassie blue. (**f**) Association of NQO1 (red) with F-actin (green) was determined by immunostaining (× 40 magnification) using a specific antibody against NQO1 and palloidin for the detection of F-actin in podocytes. Scale bar is 50 μm.

We finally studied whether NQO1 can directly regulate actin polymerization via an in vitro actin polymerization assay using pyrene conjugated G-actin which forms pyrene F-actin when it polymerizes. Actin polymerization buffer (PB) stimulated rapid polymerization of actin. Treatment with recombinant NQO1 proteins enhanced actin polymerization (Fig. 7A). Next, we performed actin polymerization assay using different concentrations of NQO1. We found that 0.5 μM slightly stimulated actin polymerization over time. However, 2 μM of NQO1 protein led to a maximum intensity of pyrene fluorescence (Fig. 7B). NQO1 activities can be inhibited by the specific NQO1 inhibitors dicoumarol and ES936, by competing with cofactors such as NADH and NADPH or by catalytic inactivation of NQO1, respectively. However, treatment with the two inhibitors did not block the NQO1-mediated actin polymerization (Fig. 7C). To further address this issue, we purified NQO1 protein from a mammalian cell line using a Strep pulldown system. Consistent with the results obtained using the recombinant protein, NQO1 protein purified from mammalian cells also promoted actin polymerization, suggesting that NQO1 is involved in actin polymerization (Fig. 7D). To investigate whether NQO1 can bind to actin filaments, we performed actin co-sedimentation experiments. NQO1 protein was found in the supernatant, but not in the pellet, indicating that NQO1 is not associated with mature F-actin (Fig. 7E). Although we did not detect direct binding of NQO1 with F-actin, co-staining data showed that NQO1 was strongly colocalized with F-actin (Fig. 7F). The colocalization of NQO1 with F-actin was eliminated by knockdown of NQO1 by treatment of siRNA for NQO1 (Fig. 7F).

## Discussion

The present study was performed to investigate the potential roles of NQO1 in the pathogenesis of DN. Previously, NQO1 has been demonstrated to have anti-oxidant properties and a protective role against kidney damage induced by I/R or cisplatin^27,28^. In the current work, a STZ-induced diabetic mouse model was used to demonstrate the roles of NQO1. To the best of our knowledge, this is the first study to demonstrate that knockout of NQO1 accelerates proteinuria and pathological changes of STZ-induced DN in mice. Furthermore, this study showed that NQO1 has an important role in the restoration of podocyte cytoskeleton architecture under diabetic conditions.

NQO1 is a representative phase II detoxification enzyme regulated by the transcription factor NRF2 in response to oxidative stress^29,32^. Oxidative stress has been recognized as a risk factor and mechanistic contributor to DN in humans and animal models^33^. Accumulated results suggest that elevated blood glucose in diabetic patients results in the generation of significant ROS that are key players in cell death^34^. Here, we found that NQO1 expression was upregulated in whole kidneys of mice with DN and cells cultured in high-glucose condition. Mechanistically, we could expect that oxidative stress is one of the contributors to the acerbated renal damages in NKO mice with DN because we have previously observed that cisplatin-induced nephrotoxicity in NKO mice was associated with increased ROS production^28^. However, ROS generation and gene expression of anti-oxidant enzymes in whole kidneys in response to STZ-induced hyperglycemia was not affected by NQO1 deficiency, suggesting that oxidative stress is not a major contributor to the development of renal injury in NKO mice with DN.

It is noteworthy that the distribution of synaptopodin in the glomerulus was abnormal in the kidneys of NKO mice under normal or hyperglycemic conditions. However, despite similar number of podocytes in NKO mice and WT mice, synaptopodin fluorescence intensity in the podocytes of NKO mice was weak, granulated, and fragmented, while that in podocytes of WT mice was strong and diffuse. Previous studies indicating that synaptopodin associates with F-actin^35,36^, suggest that the abnormal distribution of synaptopodin in NKO mice might be caused by abnormal reorganization of actin cytoskeleton. Previous studies have shown that actin filament in the FP of podocytes become disordered, short, and branched in pathological conditions such as DN^20,37^. The association of human genetic mutations in actin-related genes such as *ACTN4* (encoding actinin-4 protein)^38^, *CD2AP* (encoding CD2-associated protein)^39^, *INF2* (encoding inverted formin-2 protein)^40^, and *MYO1E* (encoding myosin IE protein)^41^, with a pathogenesis of the nephrotic syndrome, has indicated the importance of actin cytoskeleton regulation. In our study, we found that recombinant and purified NQO1 proteins can efficiently stimulate actin polymerization in vitro. Furthermore, the staining intensity of F-actin was dramatically reduced, and the staining pattern was also altered in the kidney glomerulus of NKO mice, while WT mice showed strong and diffuse staining patterns in both healthy and disease conditions. These results suggest that NQO1 contributes to actin reorganization induced by the pathogenic conditions via the regulation of actin polymerization. However, we could not observe any direct interaction with F-actin in a co-sedimentation assay. Although we could not show the exact mechanism of the regulation of actin cytoskeleton, NQO1 might initiate actin polymerization via binding with other key molecules related to actin polymerization, regulating redox reactions, or participating in the nucleation step. Recent studies have shown that the redox reaction catalyzed by Mical can regulate F-actin assembly and disassembly^42,43^. Mical is a cytosolic flavoprotein containing a redox enzymatic domain and, like NQO1, uses the pyridine nucleotide NADPH as a cofactor for its redox reaction^44^. Therefore, redox regulation by NQO1 might be one of the possible mechanisms of NQO1-mediated actin polymerization. However, the effects of redox changes induced by NQO1 on actin regulation still need to be investigated in the future. We also found elevated phosphorylation of cofilin in the kidney of NKO mice with DN and in NQO1-silenced podocytes. Cofilin is a small actin-binding protein that accelerates actin dynamics through severing of F-actin^45^. This result further supports our hypothesis that NQO1 has a critical role in the process of actin polymerization in podocytes. However, there are no evidence how phosphorylation of cofilin is regulated by NQO1 because we were unable to identify any signaling molecules related to cofilin phosphorylation following a search for a NQO1 binding protein via LC/MS/MS mass spectroscopy analysis (data not shown). Furthermore, LIMK1 in kidney from NKO mice is not different compared with WT mice. Therefore, increased phosphorylation of cofilin in NKO mice and NQO1 deficient cells might be an indirect effect of impaired actin dynamics, but not direct consequence.

It has been well known that C57BL/6 mice are typically resistant to STZ-induced DN^46^. Although WT mice showed alterations in several pathogenic parameters including mild glomerular injury, abnormal FP width, and GBM thickness under hyperglycemic conditions, still maintained normal ACR, suggesting that C57BL/6 mice are resistant to STZ-induced DN. Therefore, NKO mouse might be a useful animal model for investigating the pathogenesis of DN. Furthermore, in the current study, we identified NQO1 as a new regulator of the reorganization of impaired actin cytoskeleton in DN. The small compound Bis-T-23, which can increase actin polymerization via actin-dependent dynamin oligomerization in injured podocytes, is recognized as a potential therapeutic strategy to improve renal health in diverse kidney diseases^47^. Therefore, targeting of NQO1 might be an attractive therapeutic strategy for the treatment of DN.

## Methods

### Antibodies and reagents

Antibodies against phospho-cofilin, total-myosin light-chains (MLC), α-tubulin, LIMK1, and phospho-MLC were purchased from Cell Signaling (Beverly, MA, USA). Antibodies against NQO1 (for immunostaining), β-actin, zonula occludens-1 (ZO-1), nephrin, α-actinin-4, Wilms’ tumor 1 (WT1), CD2-associated protein (CD2AP), podocin, gp91phox (NOX2), NOX4, gp67phox, gp22phox, and total-cofilin were obtained from Santa Cruz Biotechnology (Dallas, TX, USA). Antibodies against synaptopodin were purchased from Novus Biologicals (for western blot, Centennial, CO, USA) or Progen (for immunostaining, Wayne, PA, USA). Alexa 546 anti-rabbit and Alexa 488 anti-mouse IgG were obtained from Thermo Fisher Scientific (Waltham, MA, USA). Streptozotocin (STZ) was purchased from Sigma-Aldrich (St. Louis, MO, USA). Recombinant human NQO1 protein was obtained from R&D systems (MN, USA).

### Animal models

Wild-type (WT) and NKO male mice^48^ on C57BL/6N genetic background were housed at a constant temperature (20–22 °C) on a 12:12 h light: dark schedule, with free access to food and water, under specific pathogen free conditions. Littermates of NQO1 KO mice were used as WT control mouse. For the DN model, STZ was dissolved in citrate buffer (0.1 M; pH 4.5) and prepared immediately before use. Age-matched 8-week-old WT and NKO male mice were administered STZ (50 mg/kg body weight, intraperitoneal injection) after 4 h fasting, for five consecutive days. The control mice were injected with the same volume of citrate buffer. Body weight was monitored during the duration of the experiments and blood glucose was determined using a glucometer (Accu-Chek, Roche). Mice were euthanized 8 weeks after STZ. Samples of the kidneys were collected for histology, immunohistochemistry, immunofluorescence staining, western blot, and real-time PCR analyses. NKO and WT mice were intraperitoneally injected with LPS (15 mg/kg body weight) for 1 day. Urine samples were harvested from mice maintained in metabolic cages for 4 h. All animal experiments were approved by the Institutional Animal Care and Use Committee (KRIBB-AEC-19196) and were performed in accordance with the institutional guidelines of the Korea Research Institute of Bioscience and Biotechnology.

### Albumin-to-creatinine ratio measurement

Urinary albumin excretion was measured using an Albumin (Mouse) Eliza Kit (ALPCO, NH, 03079, USA), according to the manufacturers’ instructions. Urine creatinine was measured using the chemistry autoanalyzer Toshiba 200FR (Toshiba Medical Systems Co., Tokyo, Japan). The ratio of albumin (μg/L) to creatinine (mg/L) was calculated.

### Cell culture and differentiation

Conditionally immortalized mouse podocytes were cultured as described previously^49^ in RPMI 1640 medium (Gibco-BRL, Carlsbad, CA, USA) supplemented with 10% fetal bovine serum and antibiotics. For propagation, the podocytes were cultured on type I collagen (Sigma-Aldrich) at 33 °C and the culture medium was supplemented with 10 U/ml recombinant interferon-γ to enhance t-antigen expression. To induce differentiation, the podocytes were maintained in DMEM (Gibco-BRL) supplemented with 5% fetal bovine serum on type I collagen at 37 °C without interferon-γ. Differentiation of podocytes grown for 14 days at 37 °C was confirmed by immunocytochemical staining for synaptopodin. The podocytes were grown to near confluency and were serum-deprived for 24 h prior to use. Podocytes were transfected with 20 nM siRNA specific for NQO1 or with a scrambled siRNA control (Thermo Fisher Scientific) using the transfection reagent Lipofectamine RNAiMAX (Thermo Fisher Scientific) according to the manufacturer’s instructions. Overexpression of NQO1 was achieved by transfection with a plasmid encoding NQO1 cDNA using the transfection reagent Lipofectamine LTX Plus (Thermo Fisher Scientific). Each siRNA/Overexpression-Lipofectamine mixture was then added to the cells and incubated overnight at 37 °C in a 5% CO_2_ atmosphere. After washing, the podocytes were incubated with normal culture medium. The cells were harvested for protein extraction 72 h after transfection.

### Renal histologic analysis

Renal tissues were fixed in 10% neutral buffered formalin, embedded in paraffin and each sections (4 μm) were prepared. Kidney sections were stained with hematoxylin & eosin (H&E) and periodic acid-Schiff (PAS). Glomerular size per kidney tissue was calculated from the H&E staining. Over fifty glomeruli per sample stained with PAS were scored by 2 blinded observers using the following system: 0, normal glomerular structure; 1, increased mesangial matrix deposition and hypercellularity with some loss of capillary loops; 2, increased matrix deposition and focal areas of sclerosis; 3, > 50% of glomerulus sclerosis with very few capillary loops; 4, > 75% of glomerulus sclerosis and the presence of glomerular epithelial hyperplasia lesions^50^. An average injury score was obtained for each kidney.

### Transmission electron microscopy

Transmission electron microscopy was performed as described previously^51^. Briefly, tissues were fixed with 1% glutaraldehyde in phosphate-buffered saline (PBS) for 2 h at 4 °C, and then embedded in EMbed-812 resin (Electron Microscopy Sciences, Hatfield, PA, USA). The samples were observed using a transmission electron microscope (H-7650; Hitachi, Tokyo, Japan). The foot process width was assessed by measuring the number of filtration slit membranes per 100 mm of glomerular basement membrane.

### Immunofluorescence staining

For immunofluorescence (IF) staining, 4 μm formalin fixed sections were deparaffinized, rehydrated, processed for antigen retrieval, and then incubated with the primary monoclonal antibodies against NQO1, Wilms tumor 1 (WT1), or Synaptopodin at 4 °C overnight. The sections were washed with PBS, incubated with diluted Alexa Fluor-conjugated secondary antibodies (Thermo Fisher Scientific) for 1 h at room temperature in the dark, stained with DAPI (1 μg/ml, Sigma-Aldrich), and then mounted. Fluorescence images were captured using a laser-scanning confocal microscope (Nikon, C2 Plus, Melville, NY, USA). Image quantification was performed on maximum intensity projection of Z-stacks using ImageJ. For F-actin staining, frozen kidney sections (4 μm) were fixed in 4% paraformaldehyde and permeabilized in 0.1% Triton X-100 for 10 min. The sections blocked with 5% normal goat serum for 1 h were incubated with ActinGreen 488 (Thermo Fisher Scientific) for 1 h at room temperature and DAPI. Stained sections were washed with PBS and mounted. Mounted sections were imaged by using a laser-scanning confocal microscope (Nikon, C2 Plus). The F-actin in podocytes was stained with phalloidin according to standard protocol.

### RNA isolation and quantitative real-time polymerase chain reaction

Total RNA from Kidney cortex region was isolated using TRIzol reagent (Thermo Fisher Scientific) according to manufacturer’s instructions^52^. Double-stranded cDNA synthesis was prepared by using the iScript cDNA Synthesis Kit (BioRad, Hercules, CA, USA). The resulting cDNA was subjected to quantitative real-time PCR using the StepOnePlusTM Real-Time PCR system (Applied Biosystems, Foster City, CA, USA) with AccuPower 2 × Greenstar qPCR Master Mix (Bioneer, Daejeon, Korea) according to the standard protocol. The primer sequences for each genes are as follows: for NQO1, forward, 5′-GCCCGCATGCAGATCCT-3′ and reverse, 5′-GGTCTCCTCCCAGACGGTTT-3′; AhR, forward, 5′-GCAGGATTTGCAAGAAGGAG-3′ and reverse, 5′-ACTGCTGAAAGCCCAGGTAA-3′; NRF2, forward, 5′-GGCCTTTTTTGCTCAGTTTCA-3′ and reverse, 5′-ATGTGGGCAACCTGGGAGTA-3′; HO1, forward, 5′-CAGCCCCACCAAGTTCAAAC-3′ and reverse, 5′-GGCGGTCTTAGCCTCTTCTGT-3′; catalase, forward, 5′-CGACCAGGGCATCAAAAACT-3′ and reverse, 5′-ATTGGCGATGGCATTGAAA-3′; MnSOD, forward, 5′-CCACACATTAACGCGCAGAT-3′ and reverse, 5′-TCGGTGGCGTTGAGATTGT-3′; Gpx, forward, 5′-TCCGGGACTACACCGAGATG-3′ and reverse, 5′-TTCTCCTGGTGTCCGAACTGA-3′. Gene expression were normalized to that of 18S rRNA.

### ROS measurements

For kidney extract preparation, kidney tissues were fractionated via homogenization with a tight-fitting pestle in 0.25 mol/L sucrose buffer^53^. The homogenates were centrifuged at 600×*g* for 10 min to remove the nuclear fraction, and the remaining supernatant was centrifuged at 10,000×*g* for 20 min to obtain the mitochondria pellet. The remaining supernatant was centrifuged at 100,000×*g* for 1 h to obtain the cytosolic supernatant. ROS levels were determined as a previous report^28^. Briefly, kidney tissue extracts (100 μl) were incubated with 20 μM DCF-DA and the fluorescence was recorded. To determine H_2_O_2_ levels, kidney extracts were incubated with 20 μM anokex red (Sigma-Aldrich) and 0.1 U/ml horseradish peroxidase (HRP, Sigma-Aldrich) and the fluorescence was recorded at 530 nm (excitation) and 620 nm (emission). Finally, to estimate O_2_^−^ level, the extracts were incubated with 20 μM dihydroethidium (Sigma-Aldrich) and the fluorescence was measured at 485 nm (excitation) and 620 nm (emission). All reactions were incubated at 37 °C for 60 min. Fluorescence intensity was recorded using a Victor3 Multilabel Counter (PerkinElmer, Waltham, MA, USA) and was normalized by protein concentration.

### Western blotting

Kidney tissues (cortex) or cell lysates were homogenized in RIPA (50 mM Tris–HCl, 150 mM NaCl, 1% NP40, 0.5% Na-deoxycholate, and 0.1% SDS) buffer with protease inhibitor cocktail (Roche Applied Science, Penzberg, Germany)^52^. Protein concentration was determined by using the Bradford assay. The proteins were separated via a 10 ~ 12% SDS–polyacrylamide gel electrophoresis, transferred to polyvinylidene fluoride membrane and detected with the corresponding primary and secondary antibodies.

### Strep-tagged protein purification

HEK293T cells (ATCC, VA, USA) were transfected with the strep vector or strep-tagged NQO1 plasmid by Lipofectamine LTX reagent (Thermo Fisher Scientific) and lysed with RIPA buffer containing protease and phosphatase inhibitor Cocktail (Roche Applied Science). Strep beads (IBA GmbH, Göttingen, Germany) were added to the lysate and incubated overnight at 4 °C. Beads were collected by centrifugation and washed 5 times with RIPA buffer. After incubation of beads with elution buffer (biotin 5 mM) for 30 min at 4 °C, the supernatant was transferred to filtration column (10 kDa molecular-mass cut-off; Merck, Darmstadt, Germany) and centrifuged at 13,000 rpm for 20 min to concentrate the proteins.

### Actin polymerization assay

Assays were performed using an Actin Polymerization Biochem Kit (Cytoskeleton, Denver, CO, USA) according to the manufacturer’s instructions. In a Nunc 96-well black flat bottom microplate (Thermo Fisher Scientific), pyrene G-actin mixed with recombinant NQO1 protein, purified strep-NQO1 or inhibitors (Dicumarol; Calbiochem, San Diego, CA, USA, ES936; Sigma-Aldrich) as indicated in the figures. Polymerization and polymerization kinetics were monitored using Victor X3 multilabel plate reader (10 s interval, excitation wavelength: 360 nm, emission wavelength: 430 nm; PerkinElmer).

### Actin cosedimentation assay

Actin cosedimentation assay was performed using Actin Binding Protein Biochem Kit (Cytoskeleton, CO, USA) following the manufacturer’s instructions. Briefly, recombinant NQO1 protein (R&D system, Minneapolis, MN, USA) positive control α-actinin, and negative control BSA were incubated with F actin for 30 min followed by ultracentrifugation at 150,000×g for 90 min at 24 °C. Supernatants and pellets were subjected to 12% SDS (wt/vol) poly acrylamide gel electrophoresis, and the proteins were stained with Coomassie blue.

### Statistical analysis

Statistical analyses were performed using GraphPad Prism software. All data are presented as mean ± SEM. Fluorescence intensity and immunoblot band density were analyzed using ImageJ software (1.43u, https://imagej.nih.gov). Two group comparisons were performed using two-tailed Student’s *t*-test. A *P*-value of < 0.05 was considered statistically significant.

## Supplementary information

Supplementary Figures.
