# Supplementary material for: The potential roles of NAD(P)H:quinone oxidoreductase 1 in the development of diabetic nephropathy and actin polymerization
Source: Sci Rep. 2020 Oct 20;10:17735. doi: 10.1038/s41598-020-74493-z (PMC7576596; doi:10.1038/s41598-020-74493-z)
Supplement: Supplementary file 1 — Supplementary Figures. [file 41598_2020_74493_MOESM1_ESM.pdf]

Supplementary information

NAD(P)H:quinone oxidoreductase 1 has a protective role against diabetic nephropathy and regulates actin polymerization

Sung-Je Moon[1, 2, #], Jin Young Jeong[3, #], Jae-Hoon Kim[1, #], Dong-Hee Choi[1], Hyunsu Choi[4], Yoon-Kyung Chang[5], Ki Ryang Na[3], Kang Wook Lee[3], Chul-Ho Lee[1,2, *], Dae Eun Choi[3,*], & Jung Hwan Hwang[1, 2, *]

[1]Laboratory Animal Resource Center, Korea Research Institute of Bioscience and Biotechnology (KRIBB), 125 Gwahak-ro, Yueong-gu, Daejeon 34141, South Korea

[2]KRIBB School of Bioscience, Korea University of Science and Technology (UST), 217 Gajeong-ro, Yuseong-gu, Daejeon 34113, South Korea

[3]Department of Nephrology, School of medicine, Chungnam National University, Daejeon, South Korea

[4]Clinical Research Institute, Daejeon St. Mary Hospital, Daejeon, South Korea

[5]Department of Nephrology, Catholic University of Korea, Seoul, South Korea

Fig. S1

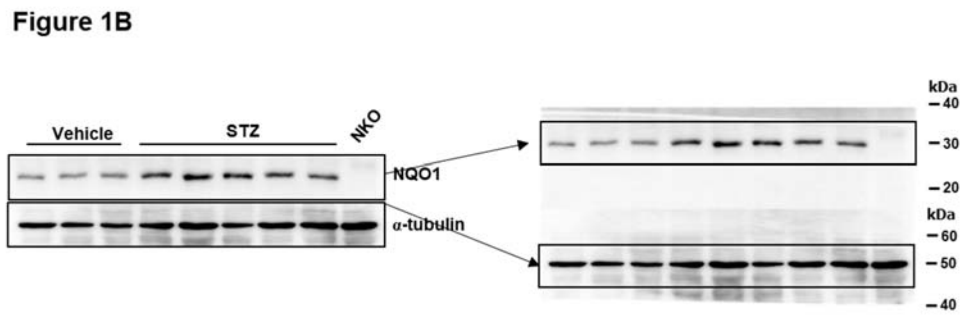

Supplementary Fig. 1. Full length blots of NQO1 and β-actin shown in Fig.1B.

Fig. S2

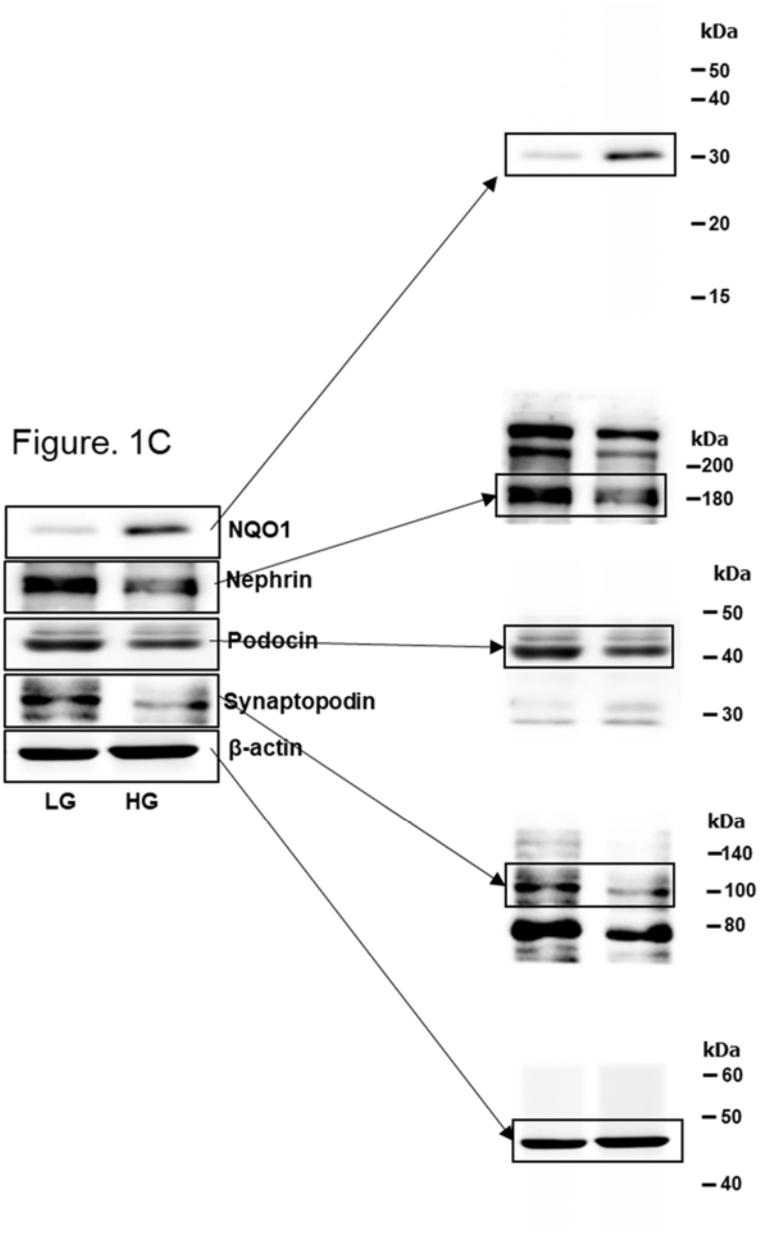

Supplementary Fig. 2. Full length blots of NQO1, Nephrin, Podocin, Synaptopodin, and β-actin shown in Fig.1C.

Fig. S3

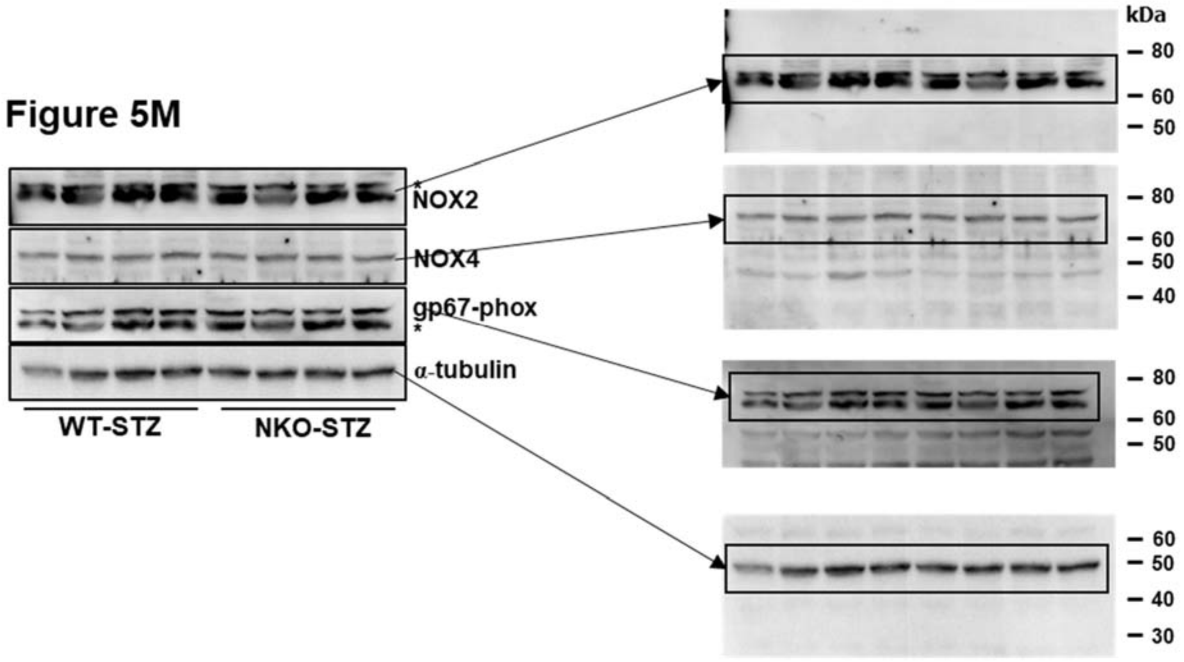

Supplementary Fig. 3. Full length blots of NOX2, NOX4, gp67-phox, and α-tubulin shown in Fig.1B.

Fig. S4

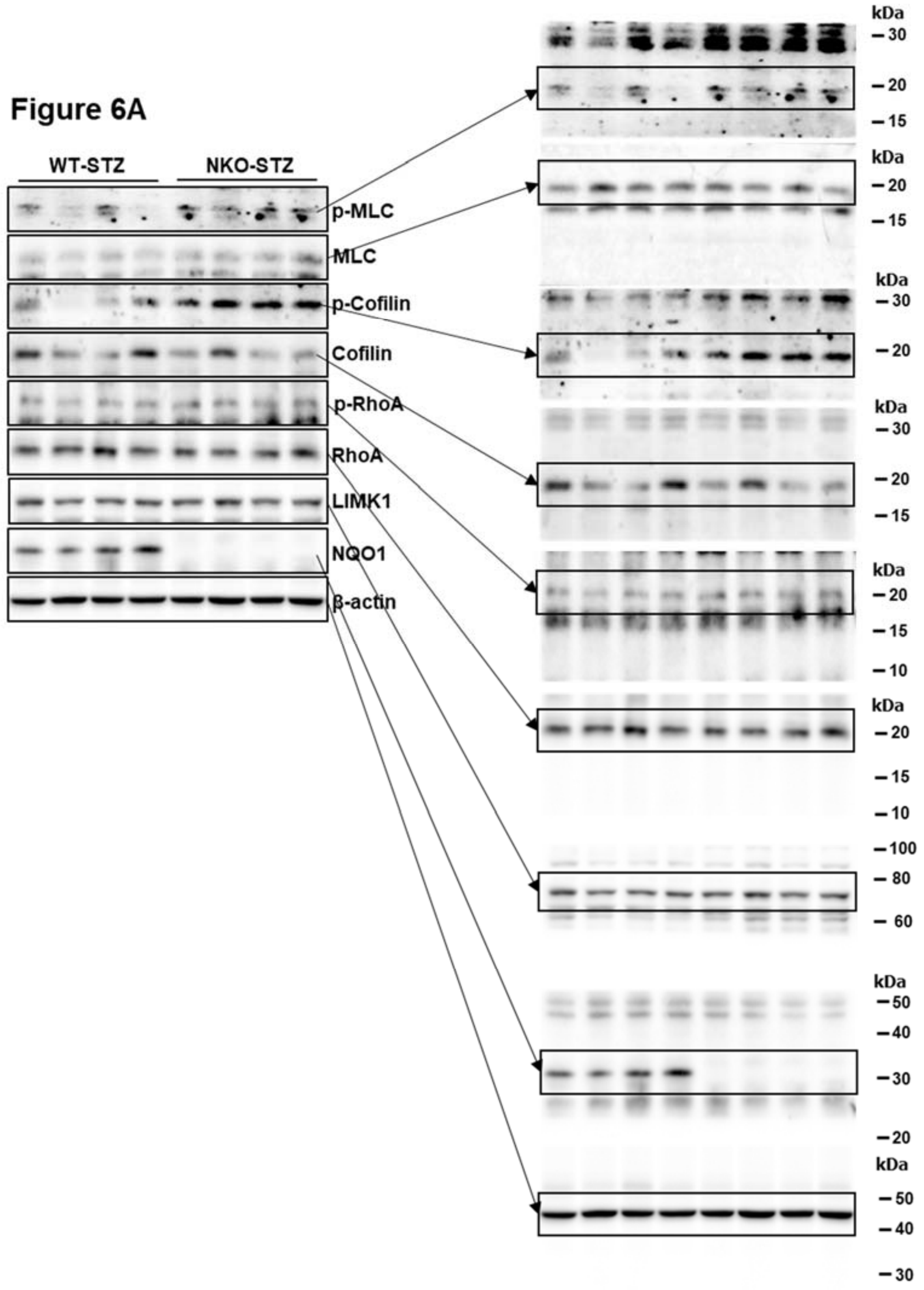

Supplementary Fig. 4. Full length blots of p-MLC, MLC, p-Cofilin, Cofilin, p-RhoA, RhoA, LIMK1, NQO1, and β-actin shown in Fig.6A.

Fig. S5

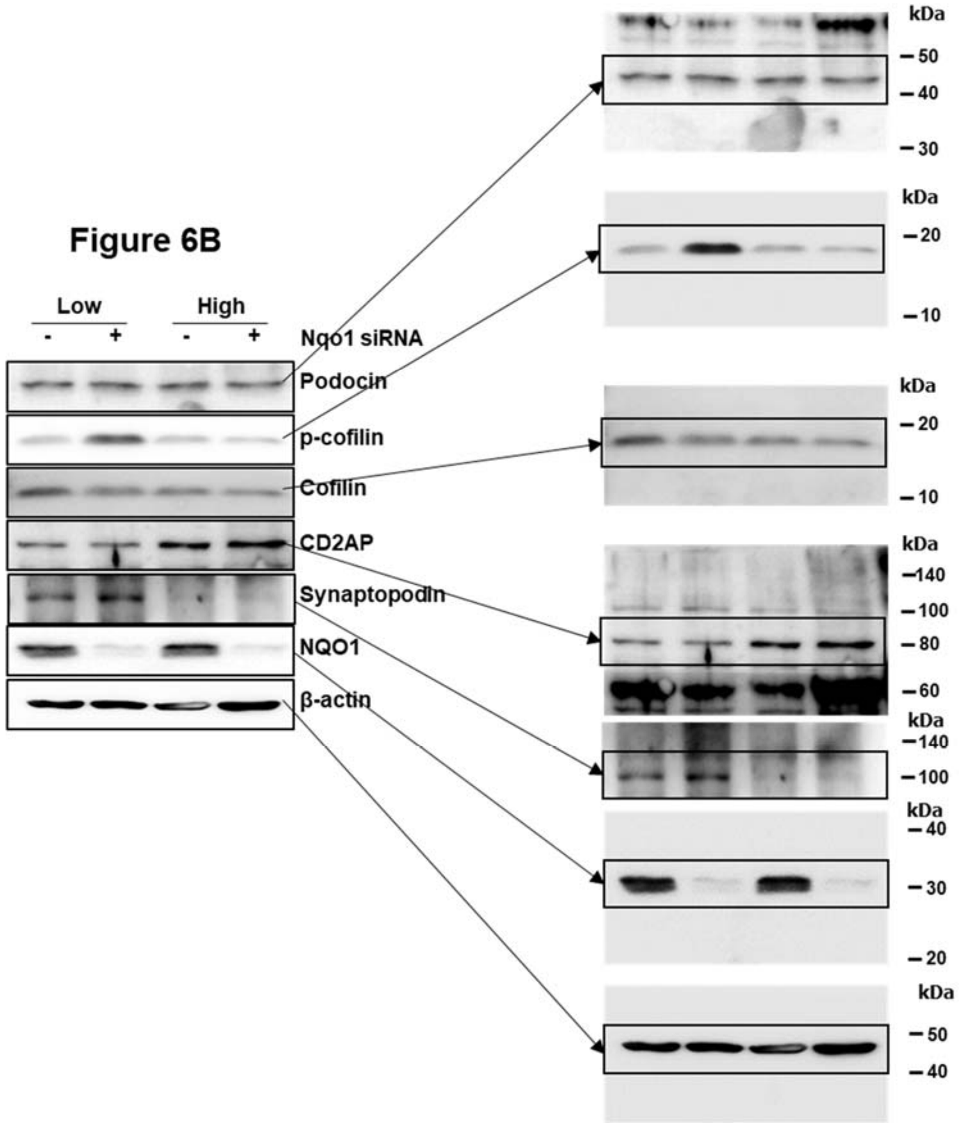

Supplementary Fig. 5. Full length blots of Podocin, p-Cofilin, Cofilin, CD2AP, Synaptopodin, NQO1, and β-actin shown in Fig.6B.
